# Response Surfaces Method and Artificial Intelligence Approaches for Modeling the Effects of Environmental Factors on Chlorophyll a in *Isochrysis galbana*

**DOI:** 10.3390/microorganisms11081875

**Published:** 2023-07-25

**Authors:** Linlin Zhang, Jie Liu, Xin Shen, Shuangwei Li, Wenfang Li, Xinfeng Xiao

**Affiliations:** College of Chemistry and Environment Engineering, Shandong University of Science & Technology, Qingdao 266510, China

**Keywords:** chlorophyll a, condition optimization, RSM, artificial intelligence algorithms

## Abstract

This study reported the condition optimization for chlorophyll a (*Chl a*) from the microalga *Isochrysis galbana*. The key parameters affecting the *Chl a* content of *I. galbana* were determined by a single-factor optimization experiment. Then the individual and interaction of three factors, including salinity, pH and nitrogen concentration, was optimized by using the method of Box–Benhnken Design. The highest *Chl a* content (0.51 mg/L) was obtained under the optimum conditions of salinity 30‰ and nitrogen concentration of 72.1 mg/L at pH 8.0. The estimation models of *Chl a* content based on the response surfaces method (RSM) and three different artificial intelligence models of artificial neural network (ANN), support vector machine (SVM) and radial basis function neural network (RBFNN), were established, respectively. The fitting model was evaluated by using statistical analysis parameters. The high accuracy of prediction was achieved on the ANN, SVM and RBFNN models with correlation coefficients (R^2^) of 0.9113, 0.9127, and 0.9185, respectively. The performance of these artificial intelligence models depicted better prediction capability than the RSM model for anticipating all the responses. Further experimental results suggested that the proposed SVM and RBFNN model are efficient techniques for accurately fitting the *Chl a* content of *I. galbana* and will be helpful in validating future experimental work on the *Chl a* content by computational intelligence approach.

## 1. Introduction

Microalgae are a group of unicellular organisms and play a key role in the food web. Some of them have been utilized as aquatic animal baits, chemical raw materials and human health foods [1,2]. The haptophyte *Isochrysis galbana* is a well-known algal species of commercial interest because it can synthesize large amounts of lipid, protein, carbohydrate and other bioactive compounds, particularly long-chain polyunsaturated fatty acids (PUFAs), eicosapentaenoic acid (EPA) and docosahexaenoic acid (DHA) [3]. Therefore, *I. galbana* has been one of the promising food sources for bivalves, larvae, crustaceans and fish in mariculture [4]. In addition, *I. galbana* can grow in extreme environmental conditions and has great potential in the treatment of wastewater with low heavy metal concentration [3,4].

Generally, microalgae cell growth and chlorophyll production are modulated by changing the culture conditions, such as temperature, light, salinity, pH and inorganic salts [5,6,7]. Thus, screening the optimal growth pattern of *I. galbana* is the crucial step for regulating the growth and photosynthesis of microalgae. Salinity is a key driver of microalgal productivity and pigment because it can change the chlorophyll, fatty acid, and other physiological and biochemical indexes of microalgae. Higher salinity hinders the normal physiological and metabolic activities by inducing osmotic pressure of microalgae cells, causing cell shrinkage and consequent damage of cell or cell components [8,9]. For most microalgae species, the optimal pH growth range is between 7 and 9. It was found that microalgae had an above 20–25% increase in growth at the pH of 7.5 and 8.0 compared with the growth at lower pH values [10]. Nitrogen is an indispensable nutrient factor for microalgae growth and important for the photosynthesis of microalgae by participating in the synthesis of proteins, nucleic acids, and chlorophyll molecules [11]. Nitrogen deficiency is known to induce a wide variety of cellular responses in microalgae, including the reduction in the chlorophyll content [12,13].

Response surfaces method (RSM) is a useful mathematical optimization model in the analysis of multivariate quantitative processing experiments [14]. Therefore, it has been frequently performed on optimal experimental designs to optimize algal growth and pigment production [15,16,17]. Artificial Intelligence (AI) algorithms consist of several types, including boosted regression tree (BRT), artificial neural network (ANN), support vector machine (SVM) and radial basis function neural network (RBFNN), etc. These are popular and effective modeling tools for predicting process performance due to their high competence, accuracy and applications in many areas of science [18]. AI algorithms have been used to improve microalgae conversion technology or to unravel the basic working principles for microalgae [19,20]. ANN was used for modeling phytoplankton abundances and to estimate the growth of polyculture microalgae by using key variables of solar irradiance and temperature [11,12]. Furthermore, the optimization algorithm of genetic algorithms (GA) was employed to change the weight and bias of ANN in order to improve their performance and lower the mean squared error [21]. Additionally, SVM was applied in the environmental variables affecting algae growth because of its good performance in the presence of few datasets [22,23]. RBFNN was used to predict *Chl a* concentration as an indicator for future water quality changes [24]. However, no study to date has made use of AI algorithms’ capacity to simulate precisely the change of *Chl a* content for *I. galbana* under different culture conditions.

In this study, the effects of salinity, pH and nitrogen concentration on the *Chl a* accumulation were screened and RSM was applied to determine the optimum process variables. Furthermore, ANN, RBFNN and SVM models were, respectively, developed to estimate the *Chl a* concentration, and their predictive capabilities of with RSM were compared by the performance indices and coefficient of correlations.

## 2. Materials and Methods

### 2.1. Culture Condition

*I. galbana* was obtained from the Institute of Oceanography, Chinese Academy of Sciences and was inoculated into the 500 mL flask containing f/2 medium of 250 mL. The temperature and intensity of illumination were set at 23 ± 1 °C 4000 µmol PhotonPAR/m²/s and 12/12 h light-dark cycle. Three parallel samples were set in each experimental group, and the inoculation rate of algae was 10%. In order to ensure uniform growth, daily random exchange of conical bottles and shaking several times was conducted.

### 2.2. Determination of Chl a Content

The culture solutions were collected and 10 mL of mixed algal liquid was centrifuged in the centrifuge tube at 4000 rpm for 20 min. After the supernatant was removed, 5 mL 90% acetone was added into the tubes and then the solution was quickly placed in the refrigerator at 4 °C for 12 h. The absorbance of the supernatant at the wavelengths of 750 nm, 664 nm, 647 nm and 630 nm were, respectively, determined with a spectrophotometric device after centrifugation for 20 min. *Chl a* content is calculated according to Formula (1) [25]:

*Chl a* (mg/L) = 11.85 × (A664 − A750) − 1.54 × (A647 − A750) − 0.08 × (A630 − A750) (1)

### 2.3. Single Factor Experiment

The effects of salinity, pH and nitrogen concentration on the growth of *I. galbana* were studied by a single-factor optimization experiment. Three independent single-factor variables and varying concentrations of salt were added to separate algal cultures, and the salinity concentration was from 10‰ to 50‰. The pH levels ranging from 6.0 to 10.0 were set up by phosphate buffer and the concentrations of nitrogen (15.0–135.0 mg/L) were chosen by adding ammonium salt for the growth condition optimization. The algal cells were inoculated into a 500 mL conical flask and then cultured in a light incubator. After 3 days of incubation, the content of *Chl a* was determined as described above. Three independently repeated experiments were performed.

### 2.4. Response Surface Methodology Model

According to the results of the single-factor optimization experiment, three-factor and three-level experiment was designed. Salinity, pH and nitrogen concentration were regarded as independent variables and *Chl a* content was chosen as the response value. The interactive effects of these three factors on the growth of *I. galbana* were studied by the response surface design method of Box–Benhnken Design in Design-Expert 8.0. The results of the response surface were analyzed by quadratic regression, including the establishment of the regression model, analysis of variance, drawing of 3D CONTOUR and prediction of the best experimental conditions.

### 2.5. ANN Regression Model

**Figure 1:**
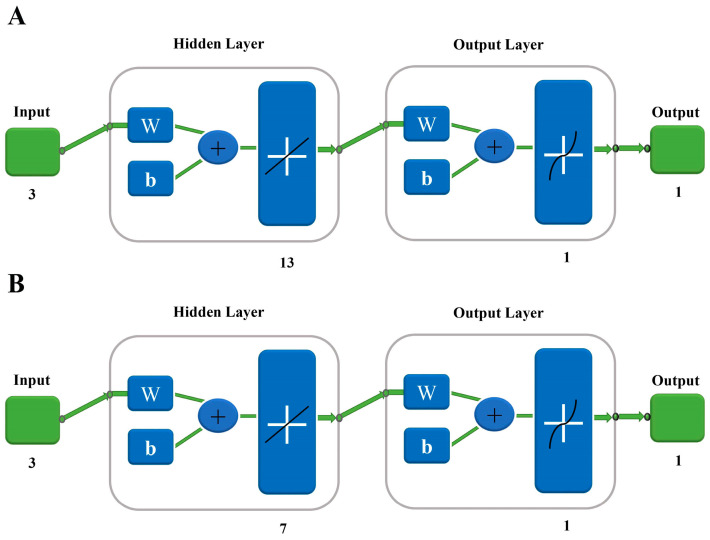
The structure of ANN (**A**) and RBFNN (**B**) for *Chl a* content prediction. W indicates the weight; b represents the bias.

ANN is one of the highly advanced methods due to its flexibility for various applications and excellent ability to deal with the complex non-linear relationships between different variables. Since the ANN neural network is very sensitive to the initial weight and threshold setting of the network, the specific parameters in the network model were assigned to the ANN according to the previous study [26]. Then the ANN model was established through the three-layer structure of 3 (input), 13 (hidden), and 1 (output) neuron layers (3-13-1) artificial neural network toolbox in the MATLAB 2016a mathematical software (Figure 1A). The input node number was the amount of salinity, pH and nitrogen concentration while the output node number was the *Chl a* content. The experimental data assessment was performed by random division with a standard approach; 80% of all data were tested in training the network, 10% of all data for validation, and 10% of all data for testing the network, to predict the best possible *Chl a* content. 

### 2.6. RBFNN Regression Model

RBFNN is based on supervised learning and is good at modeling nonlinear data. RBFNN is a special feed-forward network and requires fewer training samples with a faster training speed. To date, there has been no attempt to model the *Chl a* concentration during microalgal growth optimization using RBFNN. RBFNN consists of three layers of 3 (input), 7 (hidden), and 1 (output) neuron layers (3-7-1) and the structure is shown in Figure 1B. In this analysis, the network parameters of learning rate were set to 100. 

### 2.7. SVM Regression Model

SVM is another form of the artificial intelligence approach and can be used as an effective tool for predicting the values in many fields, including classification, regression, and time series prediction [22]. SVM also has presented a high accuracy for almost any multivariate function. Herein, the SVM model was constructed by using a software package LIBSVM toolbox (Faruto Ultimate 3.0 Version) installed in the MATLAB system [27]. The hyperplane parameters, including kernel function, epsilon, box constraint, and gamma, were opted before SVM modeling. Different hyperplane parameters were given in Table 1 and experimental data were divided into training and testing data with 13 and 4 experiment values, respectively.

### 2.8. Performance Analysis of Models 

**Figure 2:**
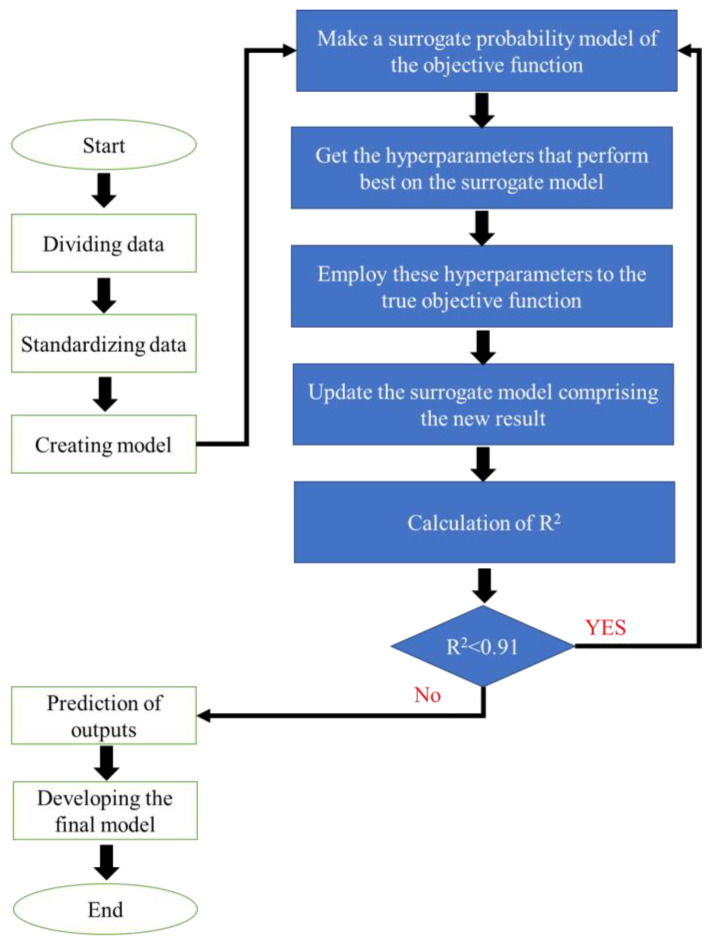
The computational flowchart of artificial intelligence models.

Herein, we developed a methodology using MATLAB to achieve high-quality numerical curve fitting by recording the number of cycles when the value of R^2^ was greater than 0.91 (Figure 2). The performance of the models was determined by employing the following indicators:

$$R^{2}=1-\frac{\sum_{i=1}^{n}{\left(Y_{p}-Y_{e}\right)}^{2}}{\sum_{i=1}^{n}{\left(Y_{p}-Y_{\mathrm{ie}}\right)}^{2}}\;\mathrm{MSE}=\overset{n}{\underset{i=1}{\sum }}\frac{{\left(Y_{e}-Y_{p}\right)}^{2}}{n}\;\mathrm{RMSE}=\sqrt{\frac{\sum_{i=1}^{n}{\left(Y_{e}-Y_{p}\right)}^{2}}{n}}\;\mathrm{MAE}=\frac{1}{n}\overset{n}{\underset{i=1}{\sum }}|Y_{e}-Y_{p}|$$

where n, Y_p_, Y_e_, Y_ie_, R^2^, MSE, RMSE and MAE represent the number of experiments, predicted value, experimental value, mean value of the experiment, coefficient of determination, mean square error, root mean square error and mean absolute error, respectively. 

## 3. Results

### 3.1. Single-Factor Experiment

#### 3.1.1. Effect of Salinity

**Figure 3:**
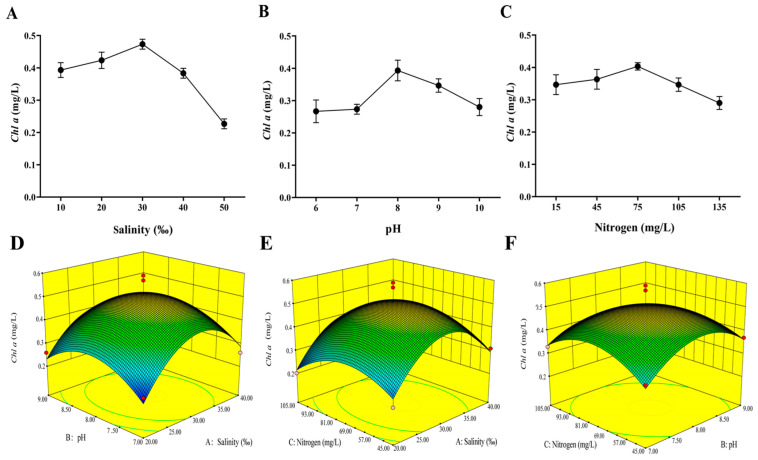
Effect of different condition factors on the *Chl a* content. (**A**): salinity; (**B**): pH; (**C**): nitrogen concentration. Response surface plots for effects of condition on the *Chl a* content. (**D**): response surface of the effects of salinity and pH on *Chl a* content; (**E**): response surface of the effects of nitrogen concentration and pH on *Chl a* content; (**F**): response surface of the effects of nitrogen concentration and salinity on the *Chl a* content.

In the process of microalgae cell growth, salinity usually changes cell osmotic pressure and affects its absorption of nutrients, subsequently leading to the change of growth and biochemical composition of microalgae [28]. The effect of salinity on *Chl a* content was tested at five different salinities (10‰, 20‰, 30‰, 40‰ and 50‰) and the result was shown in Figure 3A. *Chl a* content increased at the range of 10–30‰ and reached the maximum value of 0.47 mg/L at the salinity of 30‰. When the salinity was more than 30‰, the content of *Chl a* showed a decreasing trend. Therefore, the optimum growth salinity of *I. galbana* ranges was determined as 20‰ to 40‰. 

#### 3.1.2. Effect of pH 

The effects of variation of pH (6.0, 7.0, 8.0, 9.0 and 10.0) on *Chl a* are shown in Figure 3B. It has been observed from the figure that *Chl a* increased significantly by 44.4% at pH 8.0 with a maximum value of 0.39 mg/L compared to the values at pH 6.0. Then there was a decrease in the *Chl a* content when the pH was over 8.0. Based on these results, a pH from 7.0 to 9.0 was selected as the optimum pH range. 

#### 3.1.3. Effect of Nitrogen Concentration 

The effect of different nitrogen concentrations on the *Chl a* content was investigated and the result was presented in Figure 3C. When the nitrogen concentration was at the range of 15.0–75.0 mg/L, *Chl a* content increased with the increase in nitrogen concentration. The maximum content of *Chl a* with 0.40 mg·L^−1^ was observed at the nitrogen concentration of 75.0 mg/L and the minimum value was 0.29 mg·L^−1^ at the nitrogen concentration of 105.0 mg/L. Therefore, the optimal growth nitrogen concentration of *I. galbana* ranged from 45.0 mg/L to 105.0 mg/L. 

### 3.2. Response Surface Analysis 

Based on the results of the single-factor optimization experiment, three independent process variables, including salinity, pH and nitrogen, affect the *Chl a* content of *I. galbana*. Figure 3D presented the mutual effect of the pH and salinity, and Figure 3E presented nitrogen and salinity while Figure 3F presented the interaction of the nitrogen and pH. The response surface design and results of different environmental factors on the *Chl a* content of *I. galbana* are shown in Table 2. A quadratic multinomial regression model was obtained through the regression fitting of the experimental results:

The correlation coefficient R^2^ = 0.8935, indicated that 89.35% of the variation of *Chl a* content in response value came from selected variables. MSE and RMSE values were 0.0095 and 0.0392, respectively, suggesting that RSM has a good prediction with experiment data. Therefore, the quadratic polynomial regression equation can describe the relationship between three factors and response value.

*Chl a* (mg/L) = 507.36 − 6.41*A* + 23.08*B* − 17.61*C* − 18.30*AB* − 29.70*AC* + 4.20*BC* − 101.99*A*^2^ − 165.59*B*^2^ − 86.23*C*^2^

### 3.3. Statistical Analysis Using ANN

**Figure 4:**
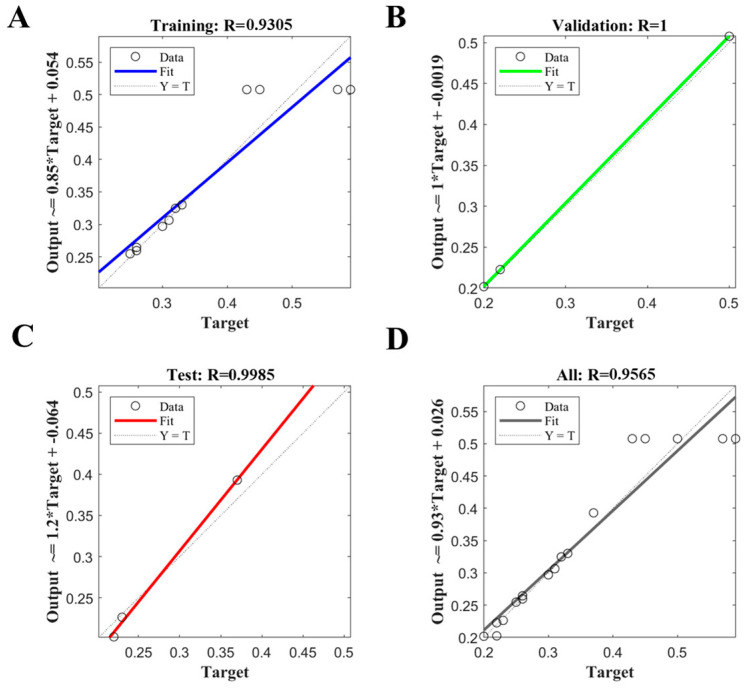
The regression plots for training (**A**), validation (**B**), test (**C**) and all (**D**) with complete data based on the ANN.

**Figure 5:**
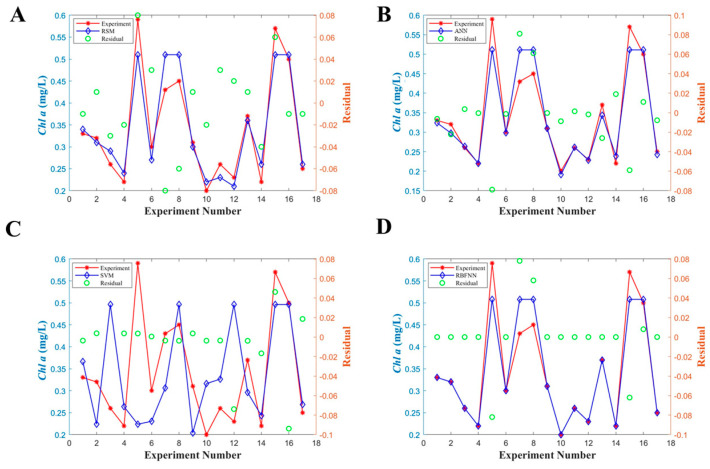
Comparison of experimental and predicted values based on the different approaches for all samples with residual. (**A**): RSM; (**B**): ANN; (**C**): RBFNN; (**D**): SVM.

Output regression coefficient values for the training, validation, test and all data with corresponding R values were 0.9305 (Figure 4A), 1.0000 (Figure 4B), 0.9985 (Figure 4C), and 0.9565 (Figure 4D), respectively. The performance of the training model based on the ANN was determined by the computation of statistical parameters in Table 3. The ANN model predicted values for R^2^ and MAE of 0.9113 and 0.0229, respectively, after 159 cycles. The observed MSE and RMSE values were 0.0087 and 0.0359, respectively, indicating that the ANN model has a higher accuracy compared with RSM (Figure 5A). Meanwhile, a correlation graph plotted between the actual values (red line) and ANN predicted values (blue line) was presented in Figure 5B. Run 5, 7 and 17 error values between experimental and ANN predicted was high value, and other run error values had less deflection of points. The maximum predicted value of *Chl a* was 0.51 mg/L at the cultural condition of salinity 30‰, pH 8.0, and nitrogen concentration with 75 mg/L.

### 3.4. Statistical Analysis Using SVM 

The SVM model was trained and its accuracy was calculated in terms of statistical parameters (Table 3). The result showed that R^2^ of 0.9127, MSE of 0.0086 and RMSE of 0.0356 was for all data with 32 cycles. Moreover, the performance of the SVM models was evaluated by analyzing the deviation of the data points with the residuals (Figure 5C). Only run 16 has a high deviation value, so it can be seen that a higher frequency of residuals was found around zero for the SVM model, confirming a remarkable agreement between predicted values and experimental values. The maximum *Chl a* content was predicted with 0.51 mg/L with the condition of salinity 30‰, pH 8.0, and nitrogen concentration 75 mg/L.

### 3.5. Statistical Analysis Using RBFNN 

The accuracy of the models developed from the RBFNN was first evaluated based on the correlation coefficient R^2^ value (Table 3). This R^2^ value reached 0.9185 after one cycle, which indicated the compatibility between the experimental and estimated values. The model was further supported by the low values of MSE and RMSE with 0.0083 and 0.0344, respectively. Additionally, it can be clearly observed that most of error values were closely similar to zero in this experiment (Figure 5D). Although the error of runs 5, 7 and 15 for the RBFNN model is quite high, the overall error is lesser as compared to RSM. Hence, it was evident that the prediction accuracy of ANN models is remarkable when simulating the concentrations of *Chl a*. 

## 4. Discussion

Microalgal growth strongly depends on cultivation factors, such as cultivation temperature, pH and nitrogen availability. Herein, a single-factor optimization experiment was used to investigate the influence of salinity, pH and nitrogen concentration on the *Chl a* content of *I. galbana*. In the previous salinity test, *I. galbana* grew in a wide range of salinities from 10 to 65‰, and 35‰ was determined as the optimal salinity [29]. The optimum growth salinity of *I. galbana* ranges in this study was 20‰ to 40‰. The difference in optimal salinity may depend on algal species and the algal products examined. For the pH, our result was similar to the previous pH range of 7.0 to 8.0 used in other studies [30,31]. For example, as the most important factor influencing *I. galbana* (T-Iso) growth rate, the optimal culture conditions occurred at pH 6.8 [32]. The optimal growth nitrogen concentration for *I. galbana* was determined as 45.0 mg/L to 105.0 mg/L. This result was consistent with the study that *I. galbana* grew very well in experimental units with 72, 144 and 288 mg/L nitrogen, which showed that nitrogen change in cultivation medium can significantly influence the content of *Chl a* [33,34]. 

The performance of models used in the present study was summarized and compared in Table 4. Among these models, ANN has been used most frequently in the fitting and regression for microalgae condition optimization compared with other AI models. In the present study, ANN had good prediction and model developing ability, with a high R^2^ value and a low MAE among all the techniques. Our result was consistent with the previously built ANN models which offered more accuracy and flexibility in modeling the non-linear conditions compared to RSM methods. For example, a structure of 4 (input), 10 (hidden), and 1 (output) neuron layers based on the ANN model was built to investigate the yield prediction of fatty acids methyl ester from exceedingly wet microalgae *Chlorella pyrenoidosa*. A higher R^2^ value of 0.94 and minimum RMSE (0.38) of the ANN model over the RSM model was observed, suggesting that ANN has better predictive ability than RSM [35]. The ANN was also used as the medium optimization for *Tetraselmis* sp. FTC209 grown under mixotrophic conditions. The trained network of 3-10-1 architecture based on the ANN produced the lowest RMSE (6.517) and a very high R^2^ (0.953), which was better in predicting the lipid productivity than RSM with R^2^ (0.922) and RMSE (10.043) [36]. 

For other models, the SVM and RBFNN were found to have good accuracy in the *Chl a* prediction with R^2^ values of 0.9127 and 0.9185, but the RBFNN had a lower MAE value and fewer cycles than the SVM model. Followingly, the RSM had an accuracy with R^2^ values of 0.8935, and a low value MAE of 0.0312. In addition, the RMSE analysis indicated that the RBFNN had the lowest value of 0.0344, followed by the SVM and ANN with 0.0356 and 0.0359, respectively. The graph of predicted values versus actual values indicated that the most of residual errors of RBFNN and SVM were closer to zero. These results represented a good fitness of these three models and the strong relation between the real and predicted *Chl a* content. Similar results were reported in the previous investigations where the RBFNN was used to analyze the predictive ability for the algal growth or blooms prediction [37,38]. The algal bloom intensification in the mid-Ganga River, India was simulated with the RBFNN model by detecting the change of in situ *Chl a* concentration values. The RBFNN model exhibited satisfactory performance with a high R^2^ value (0.9779) as well as marginally lower root mean square percent error (4.79%) and mean absolute percentage error (2.42%) [38]. The mathematical explanations of the SVM have been well-established in the previous literature. The microalga biomass productivity of *Chlorella vulgaris* was predicted by the SVM model with temperature, light-dark cycles, and nitrogen-phosphorus ratios as the independent input variables. The SVM model exhibited satisfactory performance with a low error (MAE of 0.0128 and RMSE of 0.0189) and a high R^2^ of 0.911 [18]. Additionally, the SVM model showed an excellent performance in the prediction of biodiesel production from microalgal oil of *Nannochloropsis* oculate. The predicted coefficient of determination R^2^ was 0.965 and MSE was 0.0345, and the performance improvement of 20.73% was observed with respect to the R^2^ in comparison to RSM [39]. Similarly, when artificial intelligence modeling approaches, the ANN and SVM were applied to predict CO_2_ biofixation with only 15 available experiment data, the SVM model yielded low errors of MAE (0.0128) and RMSE (0.0189), with a high R^2^ of 0.911. 

## 5. Conclusions

The optimization of cultivation parameters, like nutritional and environmental factors, are very important for microalgal growth or pigment accumulation. In the present study, the RSM with Design-Expert 8.0.6 software was adopted to identify the optimal cultural condition which enhanced *Chl a* production from *I. galbana*. The optimum culture conditions for *Chl a* concentration were determined as salinity 30‰, pH 8.0 and nitrogen concentration 72.1 mg/L, the maximum predicted value of *Chl a* content was achieved with 0.51 mg/L. Furthermore, the RSM and three different artificial intelligence models namely the ANN, RBFNN and SVM models, were used for modeling the *Chl a* content. The performances of the established models were evaluated by using indicators of R^2^, MAE, RMSE and MSE. Results suggested that the SVM and RBFNN exhibited a close alignment with experimental results with high R^2^ value and fewer cycles, demonstrating that these models had better prediction capability compared to the RSM model and were the potential option when a few data need to be processed. 

## Figures and Tables

**Table 1:** SVM initial conditions settings in the LIBSVM toolbox.

Hyperplane Parameters	Parameters	Hyperplane Parameters	Parameters
C	4	Kernel function	‘rbf’
Degree	3	Gamma	0.8
Epsilon	0.01		

**Table 2:** Analysis of variance of response surface quadratic regression model.

Source of Variance	Sum of Squares	Degree of Freedom	Mean Square	F Value	*p* Value	Salience
Model	222,500	9	24,721.72	6.53	0.0109	Significant
*A*	329.10	1	329.10	0.087	0.7767	/
*B*	4261.28	1	4261.28	1.13	0.3240	/
*C*	2480.90	1	2480.90	0.66	0.4449	/
*AB*	1339.49	1	1339.49	0.35	0.5707	/
*AC*	3529.40	1	3529.40	0.93	0.3665	/
*BC*	70.46	1	70.46	0.019	0.8953	/
*A* ^2^	43,800.30	1	43,800.30	11.57	0.0114	/
*B* ^2^	111,500	1	111,500	30.49	0.0009	/
*C* ^2^	31,304.67	1	31,304.67	8.27	0.0238	/
Residual	26,507.03	7	3786.72	/	/	/
Misfit term	5947.84	3	1982.61	0.39	0.7700	Inconspicuous
Error term	20,599.18	4	5139.80	/	/	/
Summation	249,000	16	/	/	/	/

The variance analysis of the quadratic regression model of response surface was shown in Table 2. F value of the model was 6.53, *p*-value was 0.0109 (*p* < 0.05), which indicated that the regression model was significant through software fitting, and the F value of the misfit term was 0.39, *p*-value was 0.7700 (*p* > 0.05). It was indicated that the regression model was suitable for the optimization of *I. galbana* growth. At the same time, the quadratic terms *A^2^* and *C^2^* were significant, *B^2^* was extremely significant. The regression term *p* value corresponding to this model was 0.0109 which was less than 0.05. The fitting degree of the regression equation suggested that the model was effective in predicting *Chl a* content. The maximum predicted value in *I. galbana* was 0.51 mg/L, with the corresponding cultural condition of salinity 30‰, pH 8.0, and nitrogen concentration 72.1 mg/L.

**Table 3:** Statistical parameters comparison of RSM and artificial neural network models.

Parameters	RSM	ANN	SVM	RBFNN
R^2^	0.8935	0.9113	0.9127	0.9185
MSE	0.0095	0.0087	0.0086	0.0083
RMSE	0.0392	0.0359	0.0356	0.0344
MAE Cycle number	0.0312 NaN	0.0229 159	0.0208 32	0.0169 1

**Table 4:** The predictive results of RSM and different models on the *Chl a* content.

Numbers	*A*	*B*	*C*	*Chl a* Content (mg/L)				
				Real Value	RSM	ANN	SVM	RBFNN
1	7.0	30	105.0	0.33	0.34	0.32	0.37	0.33
2	7.0	30	45.0	0.32	0.31	0.30	0.22	0.32
3	7.0	40	75.0	0.26	0.29	0.26	0.50	0.26
4	9.0	40	75.0	0.22	0.24	0.22	0.26	0.22
5	8.0	30	75.0	0.59	0.51	0.51	0.22	0.51
6	8.0	40	105.0	0.30	0.27	0.30	0.23	0.30
7	8.0	30	75.0	0.43	0.51	0.51	0.31	0.51
8	8.0	30	75.0	0.45	0.51	0.51	0.50	0.51
9	8.0	40	45.0	0.31	0.30	0.31	0.20	0.31
10	8.0	20	105.0	0.20	0.22	0.19	0.32	0.20
11	9.0	20	75.0	0.26	0.23	0.26	0.33	0.26
12	7.0	20	75.0	0.23	0.21	0.23	0.50	0.23
13	9.0	30	45.0	0.37	0.36	0.34	0.30	0.37
14	8.0	20	45.0	0.22	0.26	0.24	0.24	0.22
15	8.0	30	75.0	0.57	0.51	0.51	0.50	0.51
16	8.0	30	75.0	0.50	0.51	0.51	0.50	0.51
17	9.0	30	105.0	0.25	0.26	0.24	0.27	0.25

*A*—pH, *B*—salinity (‰), *C*—nitrogen concentration (mg/L).

## Data Availability

Not applicable.
